# Hypoxia-inducible factors as molecular targets for liver diseases

**DOI:** 10.1007/s00109-016-1408-1

**Published:** 2016-04-20

**Authors:** Cynthia Ju, Sean P. Colgan, Holger K. Eltzschig

**Affiliations:** Department of Pharmaceutical Sciences, Skaggs School of Pharmacy, University of Colorado, Auroa, Colorado 800045 USA; Department of Medicine and Mucosal Inflammation Program, School of Medicine, University of Colorado, Auroa, Colorado 800045 USA; Department of Anesthesiology and Organ Protection Program, School of Medicine, University of Colorado, Auroa, Colorado 800045 USA

**Keywords:** HIF1α, HIF2α, HIF1α, Ischemia-reperfusion liver injury, Fatty liver, Viral hepatitis, Alcoholic liver disease, Hepatocellular carcinoma

## Abstract

Liver disease is a growing global health problem, as deaths from end-stage liver cirrhosis and cancer are rising across the world. At present, pharmacologic approaches to effectively treat or prevent liver disease are extremely limited. Hypoxia-inducible factor (HIF) is a transcription factor that regulates diverse signaling pathways enabling adaptive cellular responses to perturbations of the tissue microenvironment. HIF activation through hypoxia-dependent and hypoxia-independent signals have been reported in liver disease of diverse etiologies, from ischemia-reperfusion-induced acute liver injury to chronic liver diseases caused by viral infection, excessive alcohol consumption, or metabolic disorders. This review summarizes the evidence for HIF stabilization in liver disease, discusses the mechanistic involvement of HIFs in disease development, and explores the potential of pharmacological HIF modifiers in the treatment of liver disease.

## Introduction

Acute and chronic liver diseases are a significant global public health issue. Acute liver injury due to adverse drug reactions accounts for more than half of the cases of acute liver failure and 15 % of the patients undergoing liver transplantation. Ischemia-reperfusion (IR) is another major cause of acute liver injury, which represents a key challenge in liver transplantation and significantly contributes to postoperative morbidity and mortality. Chronic liver diseases caused by viral infection, alcohol abuse, or obesity-associated metabolic disorder can develop into end-stage liver cirrhosis and liver cancer. It is estimated that in 2013 alone, there were 792,000 new cases of liver cancer and 818,000 deaths globally, with 86 % occurring in developing countries and 14 % occurring in developed countries [1].

Hypoxia-inducible factor (HIF) is a transcription factor regulating a wide range of genes involved in cellular responses to hypoxia and other tissue environmental cues. HIF is a heterodimeric complex consisting of a constitutively expressed β-subunit and an oxygen-sensitive α-subunit. To date, three isoforms of HIF-α subunit have been described, in which HIF1α and HIF2α are the best characterized. The α subunit is rapidly degraded under normoxic conditions and stabilized during hypoxia. The critical step in α-subunit degradation is the hydroxylation of proline residues by prolyl hydroxylase domain enzymes (PHD1-3), allowing recognition of the α-subunit by the von Hippel-Lindau (VHL) E3 ubiquitin ligase complex and subsequent proteasomal degradation [2, 3]. During hypoxia, PHD activity is limited by oxygen availability, leading to the stabilization of HIF-α subunit and its nuclear translocation. The α and β subunits heterodimerize and form a complex with CREB-binding protein (CBP)/p300, thereby exerting their transcriptional activity. A second hypoxic switch operates in the carboxy terminal transactivation domain of HIF-α, where hypoxia blocks the hydroxylation of Asn803, thereby facilitating the recruitment of CBP/p300 [4].

**Fig. 1:**
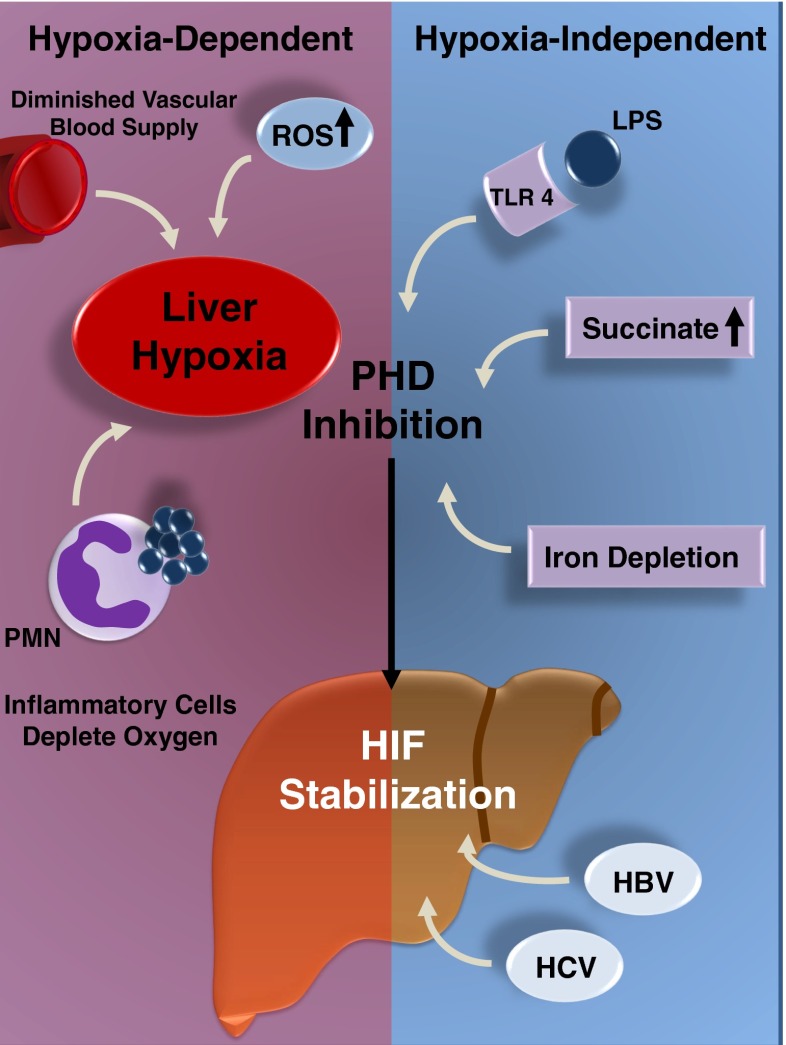
Hypoxia-dependent and hypoxia-independent mechanisms responsible for hepatic stabilization of hypoxia-inducible factor HIF. Under normoxic conditions, HIF is hydroxylated via prolyl hydroxylases (PHDs) and thereby target HIF for proteasomal degradation. Hypoxia-dependent and hypoxia-independent pathways can result in PHD inhibition and concomitant HIF stabilization during liver disease. The *left side of the figure* displays conditions of liver disease that directly lead to a hypoxic microenvironment. During conditions of liver disease, imbalance in supply and demand for metabolites—particularly oxygen—can lead to hepatic hypoxia, including diminished supply with oxygen through the vasculature. Hypoxia-associated increases in reactive oxygen species (ROS) have been reported to lead to PHD inhibition. Similarly, infiltrating inflammatory cells can deplete the microenvironment from oxygen, for example, poly-morphonuclear neutrophils (PMN) undergoing PMN burst [15]. Hypoxia-independent pathways leading to PHD inhibition include activation of toll-like receptors (particularly TLR4) through bacterial products, such as lipopolysaccharide (LPS). Accumulation of the citrate cycle intermediate succinate can function as a PHD inhibitor [181]. Iron depletion of the micro-environment for example through bacterial siderophores can lead to PHD inhibition and HIF stabilization [182]. Moreover, elevated transcription of HIF can be a direct effect during infection with hepatitis B (HBV) or hepatitis C (HCV) virus

Increased expression of HIF-1α and HIF-2α has been reported in many liver diseases, including nonalcoholic fatty liver disease (NAFLD), alcoholic liver disease (ALD), IR-induced liver injury, and hepatocellular carcinoma (HCC) [5–10]. A common feature of these liver diseases is tissue hypoxia due to an imbalance of metabolic demand and supply [11–14]. Oxygen consumption by hepatocytes and infiltrating inflammatory leukocytes is dramatically increased. For example, infiltrating neutrophils consume copious amounts of oxygen during oxidative burst. [15] In the meantime, oxygen supply is reduced due to vascular dysfunction, thrombosis, or fibrosis. Aside from hypoxia, other conditions associated with liver disease can also stabilize HIFs. For example, hepatic endotoxin levels are increased in NAFLD and ALD, and lipopolysaccharide (LPS) has been reported to stabilize HIF [16–18]. LPS-induced HIF activation is dependent on toll-like receptor (TLR)-4 and mediated through NF-κB and MAPK pathways [16, 17]. During inflammation, a TCA cycle intermediate, succinate is increased, and it functions as a HIF activator [19]. Reactive oxygen species (ROS) has also been shown to stabilize HIFs [20], and increased production of ROS is a common phenomenon in liver pathological conditions. Furthermore, evidence suggests that hepatitis B and C viruses directly stabilize HIFs under normoxic conditions (Fig. 1).

HIFs transcriptionally regulate a wide range of genes involved in cell proliferation (e.g., transforming growth factor (Tgfα) and insulin-like growth factor (Igf-2)), energy metabolism (e.g., pyruvate dehydrogenase kinase (Pdk1) and glucose transporter (Glut-1)), migration (e.g., zinc finger protein SNAI1 (Snail), twist family BHLH transcription factor (Twist1) and matrix metalloproteinases (MMPs)), and angiogenesis (e.g., vascular endothelial growth factor (Vegf) and platelet-derived growth factor (Pdgf-b)). A common response to both acute and chronic tissue injury is inflammation. HIF plays an important role in regulating innate and adaptive immune cells and their products involved in tissue inflammation. On the one hand, some studies suggest that HIFs play an important functional role in enabling innate to function in a hypoxic micro-environment. For example, bacterial killing is critically regulated by HIF1A in myeloid phagocytes [21]. Similarly, a recent study demonstrates that lipopolysaccharide-induced succinate stabilizes HIF1A and identifies succinate as a metabolite in innate immune signaling, which enhances interleukin-1β production during inflammation via HIF [19]. On the other hand, many transcriptional targets of HIFs are anti-inflammatory, for example, the extracellular signaling molecule adenosine. As such, gene-targeted mice for the Adora2b adenosine receptor are more prone to inflammation [22]. Also, many studies indicate that deletion of HIFs in models of acute or chronic inflammation is involved in a negative feedback loop dampening innate or adaptive immune responses [23–25]. As such, it is tempting to speculate that HIF could potentially function to enhance bactericidal effects of innate immune cells, while simultaneously functioning to prevent immune-mediated collateral tissue damage. These findings would indicate that HIF activators could be potent therapeutics to dampen inappropriate liver inflammation, such as occurs in the setting of ischemia and reperfusion injury [26–28].

Overall, the HIF-mediated adaptive responses to tissue hypoxia and other micro-environmental changes are critical for tissue recovery and repair from injury; and thus, HIF stabilization confers hepato-protection during acute liver damage. However, in chronic liver disease, prolonged HIF activation may be detrimental through accelerating fibrosis development, facilitating viral replication, and promoting tumor cell growth and metastasis. The following sections will (i) summarize the clinical evidence for the involvement of HIFs in a variety of acute and chronic liver diseases, (ii) discuss the current understanding and knowledge gaps of the mechanistic involvement of HIF1α and HIF2α in the disease development and progression (Table 1), and (iii) highlight the potentials of activating or inhibiting HIFs as therapeutic interventions to treat various liver diseases (Table 2).

**Table 1:** Comparing the roles of HIF1α and HIF2α in various liver diseases

Liver disease	Role of HIF1α	Role of HIF2α
I/R liver injury	Protective [34, 35, 41]	Protective [46]
NAFLD	Not clear	Contributes to steatosis [13, 77, 78]
ALD	Controversial [92, 93]	Not been studied
Liver fibrosis/cirrhosis	Pro-fibrogenic [102, 104, 105]	Not been studied
Viral hepatitis	Contributes to disease progression [14, 115, 121]	Contributes to disease progression [122, 123]
HCC	Contributes to tumorigenesis [143, 144, 154, 159]	Controversial [133, 146, 147]

**Table 2:** Examples of ongoing clinical trials related to HIF and liver diseases

Drug	Patient population	Purpose of study	Clinical trials.gov number
EZN-2968 (anti-sense oligonucleotide inhibitor of HIF-1α, NCI)	Patients diagnosed with liver cancer who have not responded to standard treatments	Determine the safety and effectiveness (Phase I)	NCT01120288 (completed)
(HIF-1α Analysis, Northwestern University)	Patients diagnosed with HCC	Compare levels of HIF-1α expression in HCC tumor explants	NCT00866957 (recruiting)
(PET, Siemens Molecular Imaging)	Cancers of head and neck, lung, liver, rectal, or cervix	PET imaging to detect tumor hypoxia regions (phase II)	NCT01075399 (completed)
RO7070179 (HIF-1α mRNA antagonist, Hoffmann-La Roche)	Patients diagnosed with HCC	Proof-of-mechanism of HIF-1α inhibition by a decrease of mRNA (Phase 1b)	NCT02564614 (recruiting)
Bevacizumab (anti-VEGF mAb, NCI)	Primary HCC, advanced HCC, localized unresectable HCC, recurrent primary HCC	(Evaluating the efficacy of combining bevacizumab with erlotinib in treating advanced HCC, Phase II)	NCT00365391 (completed)
Molecular Adorbent Recirculating System (MARS, Medical University of Vienna)	Hypoxic hepatitis, ischemic hepatitis, shock liver, hypoxic liver injury, acute liver failure	Determine whether MARS improves hepatic hemodynamics and functions in severe hypoxic hepatitis (Phase II)	NCT01690845
OXY111A (HIF-1α inhibitor, University of Zurich)	HCC, cholangiocarcinoma, pancreatic neoplasms, colorectal neoplasms	Evaluate safety and determine the maximum tolerated dose of OXY111A (Phase I and II)	NCT02528526 (recruiting)

### HIF in ischemia-reperfusion (IR)-induced acute liver damage

IR is characterized as a restriction of blood supply to an organ followed by reperfusion and re-oxygenation of blood vessels. Restriction of blood supply causes tissue hypoxia and imbalance of metabolic supply and demand [26]. Reperfusion is usually accompanied by tissue damage and inflammatory responses. IR liver injury occurs in a number of clinical settings such as hemorrhagic shock, trauma, liver surgery, and liver transplantation [29], which is the only effective treatment for end-stage liver diseases. Associated with causing liver dysfunction and even liver failure, IR liver injury represents the major challenge of liver transplantation and significantly contributes to postoperative morbidity and mortality [30]. There are two types of IR liver injury caused by warm IR and cold IR. The warm IR develops during liver transplantation surgery, shock, or trauma. The cold IR occurs during ex vivo organ preservation. The ischemia time is a good predictor of patient and graft survival. During surgery, the warm ischemic time ranging from 15 to 50 min will result in significant morbidity [31]. A meta-analysis of 26 studies revealed that both patient and graft survival declines significant when the cold-ischemia time exceeds 12.5 h [32]. It has also been reported that cold-ischemia time longer than 6 h results in nearly threefold higher risk of graft failure [33]. Due to organ shortage, currently only one third of the patients waiting for a liver transplant actually undergo the procedure (US Department of Health and Human Services. *Organ Procurement and Transplantation Network* (online), http://optn.transplant.hrsa.gov/data/ (2012). To cope with this issue, criteria for donor organs have been extended to include those from older, steatotic, and non-heart-beating donors. These “marginal” organs will be more prone to damage during procurement, preservation, and surgery and thus are particularly susceptible to IR injury.

Complex molecular and cellular mechanisms account for IR liver injury. Microcirculation dysfunction occurs at early time points as result of vasoconstriction and the swelling of sinusoidal endothelial cell and Kupffer cell. The narrowing of sinusoids entraps leukocytes, further hindering blood flow and causing tissue hypoxia. In such settings, the “master regulator” of cellular response to hypoxia, HIF, is stabilized. The HIF activation is likely an adaptive response to IR. Studies using pharmacological approaches to stabilize HIFs have revealed their protective function during IR liver injury [34–36]. The underlying mechanism of the protection involves HIF-induced transcriptional regulation of myriad of genes that regulate multiple pathways of cell adaptive responses to hypoxia, including reprogramming of cellular energy metabolism (Pdk-1, Glut-1, carbonic anhydrase (CA4/9)) and promoting angiogenesis ((Vegf, nitric oxide synthase (NOS)). Moreover, HIFs play an important role in the upregulation of cytoprotective molecules, such as heme oxygenase (Hmox-1), heat-shock proteins (HSPs), and erythropoietin. It has been reported that HIF-1α suppresses hepatocellular carcinoma cell apoptosis through upregulating Bcl-2 and inhibiting the expression and mitochondrial release of Omi/HtrA2, a serine protease involved in capase-dependent apoptosis [37]. It has also been demonstrated that HIF-1α can reduce palmitic acid-induced lipotoxicity and ER stress in hepatocytes [38]. These findings suggest that the role of HIF in promoting hepatocyte survival may be an important protective mechanism during I/R liver injury.

It has been reported that prolyl hydroxylase-1 (PHD1) negatively regulates IkappaB kinase-beta and thus NF-κB activity [39], suggesting that inhibiting PHD1 could promote cell survival through enhancing NF-κB activation. In fact, it has been shown that PHD1^−/−^ mice are less susceptible to the development of colitis due to decreased epithelial cell apoptosis and enhanced epithelial barrier function [40]. There have been two studies of the role of PHD1 in liver injury, and the findings are consistent with the notion that PHD1 inhibition promotes cell survival. One study demonstrated that PHD1^−/−^ mice were protected against acute I/R liver injury [41]. Moreover, knockdown of PHD1 in hepatocytes conferred tolerance of the cells to hypoxia due to reduced oxidative stress. Another study demonstrated that liver regeneration was significantly enhanced in PHD1^−/−^ mice compared to WT mice. This effect was due to increased hepatocyte proliferation with increases in c-myc transcriptional activity [42]. The results from both studies suggest that short-term PHD1 inhibition may be a novel therapy to attenuate or prevent I/R liver injury and to facilitate liver regeneration after surgical resection.

**Fig. 2:**
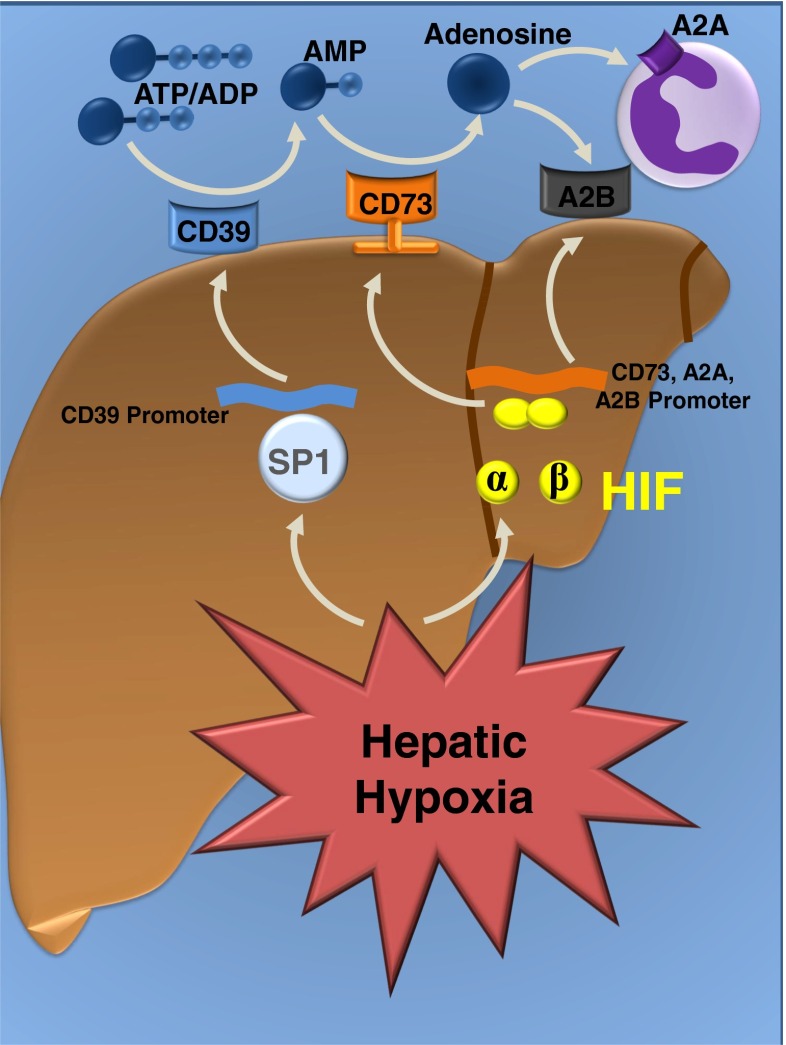
Hypoxia-stimulated adenosine production and signaling during hepatic ischemia and reperfusion injury. During liver transplantation, ischemia and reperfusion injury of the liver graft results in profound hypoxia and concomitant increases in hypoxia-dependent signaling pathways. One of the main outcomes of these transcriptional programs is increased production and signaling of the extracellular signaling molecule adenosine. During liver injury, multiple cell types release nucleotides in the form of adenosine triphosphate (ATP) or adenosine diphosphate (ADP). ATP and ADP are enzymatically converted via CD39 to adenosine monophosphate (AMP), which in turn is converted to adenosine via the enzyme CD73 [49]. Conditions of hepatic hypoxia will result in the transcriptional induction of CD39 via the transcription factor SP1 [45] and of CD73 via the transcription factor hypoxia-inducible factor HIF [47]. Similarly, the A2A and A2B adenosine receptors are transcriptionally induced by HIF [48, 49]. Many studies have implicated increased extracellular adenosine signaling in liver protection from ischemia and reperfusion injury [53]

Furthermore, hypoxia-driven increases in extracellular adenosine signaling represent an important pathway mediating the protective effect of HIFs during IR liver injury. In the extracellular compart, adenosine can function as a signaling molecule. It mainly stems from the breakdown of extracellular nucleotides and nucleosides, such as ATP, ADP, or AMP [43, 44]. Several studies have shown that during conditions of ischemia and reperfusion injury, the enzymatic system that controls extracellular nucleotide generation is transcriptionally induced, including the enzymes CD39 (ATP/ADP conversion to AMP) and CD73 (AMP conversion to adenosine) [45–47]. While CD39 is transcriptionally controlled by the transcription factor SP1, CD73 is a direct HIF target gene (Fig. 2). Moreover, other studies demonstrate that adenosine receptor signaling is increased during conditions of limited oxygen availability due to HIF-dependent induction of the A2A and A2B adenosine receptors [48, 49]. Such liver protective pathways can for instances be harnessed by hypoxic preconditioning, leading to a concomitant induction of the Adora2b adenosine receptor subtype [50], a known transcriptional target of HIF1α [51, 52]. Other studies implicate the Adora2a adenosine receptor in liver protection [53], which is a known target gene of HIF2α [54]. Again, other studies implicate HIF-controlled transcriptional repression of adenosine transporters in hypoxia-elicited adaptation and tissue protection [55, 56]. As such, many studies provide a rational for hypoxia-elicited increases in adenosine generation and signaling as an important endpoint in liver protection during ischemia and reperfusion injury (Fig. 2).

Overall, pre-clinical studies have provided strong evidence highlighting the therapeutic potential of HIF stabilization in attenuation and prevention of IR liver injury. One approach is surgical preconditioning by clamping the hepatic artery, which has been shown to sufficiently activate HIF signaling pathway and promote cell survival [57, 58]. A clinical trial of ischemic preconditioning induced by 10-min hiliar clamping demonstrated improved biochemical markers of liver function and increased HIF-1α levels [59]. However, ischemic preconditioning in a surgical transplant setting is more challenging than pharmacological activation of HIFs. As such, pre-clinical and clinical studies are on-going to explore the efficacy of HIF modifiers. Iron chelator, mangafodipir, which inhibits PHDs, has been shown to activate HIF-1α and mitigate IR liver injury in rats [34]. A PHD inhibitor, ethyl 3,4-dihyroxybenzoate, has been shown to activate HIF-1α and its target Hmox-1, thereby inhibiting mitochondrial permeability transition and attenuating IR liver injury [36]. More recently, several promising pharmacologic HIF activators are being studied in patients for the treatment of renal anemia [60]. Similar studies evaluating the beneficial effects of HIF stabilization before or during liver transplantation are urgently needed. The use of pharmacological HIF activators could represent a novel therapeutic approach to significantly dampen hepatic ischemia and reperfusion injury and thereby contributing to improved outcomes during liver transplantation. Similarly, clinical studies exploring adenosine signaling in the context of hepato-protection during liver transplantation have to be further explored.

### HIF and nonalcoholic fatty liver disease (NAFLD)

NAFLD is becoming the most common chronic liver disease worldwide due to the epidemic increase of incidence of obesity, diabetes and metabolic diseases. It is estimated that NAFLD affects 9–37 % of the population worldwide [61–63]. In the US, NAFLD affects 30 % of the general population and about 90 % of the morbidly obese individuals [64, 65]. NAFLD consists a range of disorders from simple fat accumulation in the liver (steatosis) to pathological nonalcoholic steatohepatitis. Steatosis is defined as deposition of triglycerides exceeding 55 mg per gram of liver or the presence of triglycerides droplets in more than 5 % of hepatocytes [66]. Hepatic triglycerides formation is attributable to three pathways, including diet, de novo lipogenesis, and fatty acid release from the adipose tissues. In the liver, free fatty acid is removed by oxidation in the mitochondria, stored as triglycerides lipid droplets or secreted as a constituent of very-low-density lipoproteins (VLDL). Steatosis arises from an imbalanced hepatic triglycerides acquisition and removal. For example, it is estimated that a typical American daily diet consisting 100 g of fat will result in 20 g of fat deposition in the liver, accounting for half of the total triglycerides content of an average liver. Although steatosis is benign initially, it represents a key step in the development of hepatitis with hepatocyte damage, infiltration of inflammatory leukocytes, and collagen deposition (fibrosis). In approximately 10–30 % of individuals with nonalcoholic steatohepatitis, the disease progresses into liver cirrhosis within 10 years [67]. Among liver cirrhosis patients, as high as 40–60 % will develop hepatocellular carcinoma (HCC) [68, 69].

Hypoxia occurs in the development and progression of NAFLD as result of increased metabolic demands and oxygen consumption, as well as hepatic sinusoids constriction due to hepatocyte swelling and fibrotic scar accumulation [70–72]. Hypoxia has been associated with perturbation of lipid homeostasis. Hypoxic stress of cells or tissues results in lipid accumulation [73, 74]. It is also reported that hypoxia induces genes involved in lipid synthesis, storage, and uptake [75, 76]. These findings, as well as the observation of HIF stabilization in animals and patients with NAFLD, implicate a role for HIF in regulating hepatic lipid metabolism. The majority of the published reports support the hypothesis that HIFs, HIF-2α in particular, promote hepatic steatosis.

It has been reported that deletion of both PHD2 and PHD3 in the liver results in severe hepatic steatosis with increased expression of HIFs [77]. Mice with hepatocyte-specific deletion of VHL, resulting in the overexpression of both HIF-1α and HIF-2α, also exhibit increased hepatic lipid accumulate compared with WT mice [78]. Similar to the VHL knockout mice, hepatic steatosis is also observed in another mouse model of HIF overexpression, in which HIF-1α and HIF-2α variants that cannot be hydroxylated were introduced [79]. Between the two isoforms, HIF-2α is shown to play a more important role in regulating hepatic lipid metabolism. In the PHD2/PHD3 double knockout mice, it is demonstrated that HIF-2α, but not HIF-1α, is associated with steatosis [77]. Similarly, in the VHL-deficient mice, deletion of HIF-2α, but not HIF-1α, attenuates hepatic steatosis, suggesting that HIF-2α is the main promoter of steatosis [13, 78]. Although the direct molecular targets of HIF-2α are not clear, the data demonstrate that HIF-2α-overexpressed mice exhibit increased expression of lipogenic genes (SREBP1c and FAS) and decreased expression of genes involved in fatty acid oxidation (PPARα and carnitine palmitoyl-CoA transferase-1,Cpt1) [13, 78].

Accumulating evidence suggests an important role of HIFs in the regulation of lipid metabolism and NAFLD development [80]. However, it remains unclear whether genes involved in lipogenesis, lipid storage, and fatty acid oxidation are direct transcriptional targets of HIF2α (or HIF-1α) or they are increased as an indirect effect of HIF stabilization. More detailed molecular studies to address this question are warranted to elucidate the mechanism of HIF-mediated regulation of lipid metabolism. The knowledge gained from such studies will substantiate the involvement of HIFs in the pathogenesis of NAFLD and provide the basis for developing HIF-targeted therapeutic strategies.

### HIF and alcoholic liver disease (ALD)

Alcohol abuse is a leading cause of chronic liver disease that affects millions of people worldwide. ALD encompasses a range of disorders from steatosis to alcoholic hepatitis, cirrhosis, and HCC. Alcohol-related liver cirrhosis accounts for nearly half of liver cirrhosis-associated deaths in the US [81, 82]. Among patients with alcohol-induced advanced liver cirrhosis, approximately 10 % will develop HCC [83]. Although the majority of heavy drinkers (>90 %) develop steatosis, only about one third will develop cirrhosis, suggesting the involvement of other risk factors. For example, females are more susceptible to ALD than males, probably due to estrogen, higher proportion of body fat, and lower levels of alcoholic dehydrogenase. As another risk factor, obesity accelerates cirrhosis development in ALD patients [84, 85]. Moreover, it is clearly shown that hepatitis B or C and alcohol drinking synergistically accelerate the disease progression to liver cirrhosis and HCC [86]. Aside from the direct hepatotoxic effect of ethanol metabolism, various hepatic non-parenchymal cells and their soluble products contribute to liver injury and inflammation during ALD. A better understanding of the pathogenesis is important for developing new therapies, which currently are limited to abstinence and corticosteroids.

After either acute or chronic ethanol, oxygen consumption by the liver is increased [87, 88] due to ethanol-induced hormones (e.g., catecholamine), metabolic demands, and oxidative stress [89]. The resulting liver tissue hypoxia is observed in animal models and in patients with ALD [89–91]. HIF stabilization has been reported in chronic alcoholics and in a murine model of ALD [92–94]. Alcohol-induced fatty liver shares similar clinical features and underlying pathogenesis with NAFLD. Therefore, it is likely that HIF-2α contributes to the development of hepatic steatosis during ALD as in NAFLD. However, the role of HIF-2α in the pathogenesis of ALD has not been investigated. Up to date, there are only two reports describing the involvement of HIF-1α in alcohol-associated hepatic steatosis, and their findings are contradictory.

Both studies used the HIF-1α ^f/f^/Alb^Cre^ mouse, in which HIF-1α is deleted conditionally in hepatocytes. The earlier study demonstrated that compared with WT mice, those deficient of HIF-1α developed reduced hepatic steatosis and hypertriglyceridemia [93]. Although the signaling pathway that mediates the downstream pro-steatotic function of HIF-1α was not a focus, this study demonstrated that monocyte chemotactic protein (MCP)-1 was an upstream activator that increased HIF-1α protein expression and its DNA-binding activity. In contrast, the later study demonstrated that alcohol feeding led to enhanced hepatic steatosis and serum triglycerides when HIF-1α was deleted in hepatocytes [92]. The data showed that lipogenic genes, such as acyl-CoA carboxylase (ACC)-1, fatty acid synthase (FAS), and stearoyl-CoA desaturase (SCD)-1 were expressed at higher levels in HIF-1α-deficient than WT mice. These lipogenic genes are regulated by sterol regulatory element-binding protein (SREBP)-1c, which is repressed by differentiated embryo chondrocyte 1 (DEC1). DEC1 is known to be a transcriptional target of HIF-1α. In supporting the role of DEC1 in mediating the anti-steatotic function of HIF-1α, the data demonstrated that DEC1 was elevated in alcohol-fed WT but not HIF-1α-deficient mice. Furthermore, DEC1-expressing plasmid, as well as the PHD inhibitor dimethyloxaloylglycine (DMOG), was able to ameliorate steatosis in HIF-1α-deficient mice [92].

The discordant results obtained from the two studies may be due to differences in the experimental details, such as the dose of ethanol, gut microbiome differences of animals, and the choice of control mice. Additional studies are warranted to reconcile the discrepancies and more importantly, to elucidate the molecular signaling pathways that mediate the involvement of HIFs. Furthermore, these two reported studies only focused on investigating the role of HIFs in alcohol-induced steatosis, which is an early manifestation of ALD. However, the disease progresses to much more severe forms including steatohepatitis, cirrhosis, and HCC. It is important for future studies to elucidate whether and how HIFs may play a role in the disease progression.

### HIF and liver fibrosis/cirrhosis

Chronic liver injury and inflammation caused by viral hepatitis B or C infections, alcohol abuse, NAFLD, and biliary obstruction often results in tissue fibrosis [95]. Fibrosis is part of a normal wound-healing response to tissue injury. However, repeated injury causes chronic inflammation, matrix deposition, and angiogenesis, which leads to progressive fibrosis. In some patients, liver fibrosis progresses to cirrhosis, in which hepatocytes are replaced by scar tissue with excess collagen produced by hepatic stellate cells. Advanced liver cirrhosis is characterized by portal hypertension and disruption of hepatic architecture, which often result in liver failure requiring liver transplantation [95]. As an end-stage liver disease, cirrhosis is ranked as the tenth leading cause of death in the Western world [96]. It is estimated that a 10-year mortality rate of established cirrhosis is 34–66 % [97]. Moreover, liver cirrhosis is clearly a risk factor for the development of HCC [98, 99].

Liver fibrosis causes an increased resistance to blood flow [100], which together with sinusoidal capillarization and sinusoidal obstruction syndrome causes tissue hypoxia [101]. Interestingly, hypoxic areas co-localize with those of fibrosis, suggesting a link between hypoxia and fibrogenesis [6]. Moreover, HIF stabilization has been reported in experimental models of liver fibrosis [102]. These observations suggest that HIFs may contribute to fibrosis development. In support of this hypothesis, clinical studies of human cirrhotic liver reveal that HMOX-1 and VEGF, well-known targets of HIFs, are detected in cells of fibrotic septa [6, 10, 103].

Although the role of HIF-2α has not been investigated, the pro-fibrogenenic effects of HIF-1α in multiple cell types of the liver have been reported in experimental models. CCl_4_-induced fibrosis is a commonly used model, in which mice are treated with CCl_4_ twice weekly for at least 5 weeks. In this model, liver fibrosis develops as result of chronic tissue damage and wound healing. Surgical ligation of the bile duct represents another commonly used model to induce obstructive cholestatic liver injury, which causes inflammation and leads to liver fibrosis after 21 days. It has been demonstrated that compared with WT mice, those with hepatocyte-specific HIF-1α deletion developed reduced liver fibrosis when subjected to bile duct ligation or chronic CCl_4_ treatment [102, 104]. Myeloid cell-specific deletion of HIF-1α or HIF-1β suppresses bile duct ligation-induced hepatic expression of Pdgf-b, alpha-smooth muscle actin, and type I collagen, all markers of fibrosis [105]. Hypoxic exposure of hepatic stellate cells isolated from WT and HIF-1α-deficient mice revealed that the expression of several pro-fibrogenic factors, such as Vegf, placenta growth factor, macrophage migration inhibition factor, CCR1, and CCR5, are dependent on HIF-1α [105].

HIF-induced fibrogenesis can be a direct or indirect effect of HIF transcriptional activity. Many pro-fibrogenic factors, such as Vegf, Mcp-1, plasminogen activator inhibitor-1, Pdgf-a, Pdgf-b, fibroblast growth factor (Fgf)-2, and MMPs are transcriptional targets of HIFs. Moreover, adenosine production and its receptor signaling are regulated by HIFs, and adenosine signaling plays a role in promoting liver fibrosis. It has been shown that activation of Adora2A induces collagen production by hepatic stellate cells [105], and inhibition or deletion of Adora2A suppresses liver fibrogenesis [106, 107]. The pro-fibrotic function of adenosine is also dependent on Adora2B, as its antagonist (MRS1754) has been shown to reduce liver fibrosis [108].

Accumulating evidence now suggests a tight link between angiogenesis and fibrogenesis, both occur during chronic inflammatory liver diseases. Many factors that are upregulated during angiogenesis also have profibrogenic effects, such as Vegf, Fgf2, and Pdgf. Therefore, the role of HIFs in promoting angiogenesis may indirectly promote liver fibrosis. Another indirect mechanism for the pro-fibrotic function of HIFs lies in their role in promoting inflammatory leukocytes accumulation in the liver. Infiltration of inflammatory cells require the interaction between CXCR4 and its ligand, stromal cell-derived factor-1 (SDF-1), both are HIF target genes [109]. In other tissues, it has been demonstrated that HIF-1-dependent epithelial-mesenchymal transition (EMT) contributes to fibrogenesis. The mechanism involves HIF-induced upregulation of lysyl oxidases, Snail1, and Twist expression [110, 111].

Therapy for fibrosis/cirrhosis is limited. Because of the pro-fibrotic role HIFs, therapeutic inhibition of HIF-1α and/or HIF-2α may attenuate fibrosis and slow its progression to cirrhosis and HCC. However, HIF stabilization could also improve ischemic disorders associated with cirrhosis. As such, PHD inhibitors have been proposed for the treatment of fibrosis. A recent study suggests that different HIF target genes respond differently to PHD inhibitors [77]. Thus, it is possible to design PHD inhibitors that induce erythropoietin without significantly upregulating pro-fibrotic genes. To achieve this, a better understanding of the target genes that mediate the pro-fibrotic effect of HIFs is necessary.

### HIF and viral hepatitis

Hepatitis B virus (HBV) is a member of the hepadnavirus family. HBV infection affects 350–400 million people, accounting for 5 % of the world population [112]. The majority of infected people are Asian (75 %), whereas the prevalence is low in Western countries (<1 %) [113]. Hepatitis C virus (HCV) is a single-stranded RNA virus that infects and replicates in hepatocytes. HCV infection affects 180 million people worldwide with higher prevalence in Japan, Europe, and the US than other regions [112]. As a major cause of acute and chronic liver injury, viral hepatitis often leads to serious end-stage liver diseases such as cirrhosis and HCC [114]. Chronic HBV infection has been shown to increase the risk of HCC development by 5- to 100-fold. Similarly, HCV infection is associated with 15- or 20-fold increase in the risk for developing HCC. In fact, more than 80 % of HCC patients has HBV or HCV infection [113]. Although vaccination prevents new cases, eradication of HBV in infected individuals has not been effective. Recently developed HCV protease inhibitors can efficiently eliminate the virus in the vast majority of patients. However, patients with hepatitis-associated cirrhosis remain at high risk of developing HCC. Liver transplantation is usually required once HCC occurs; however, with a high cancer recurrent rate, “re-transplantation” is necessary. Therefore, it remains important to identify new therapeutic targets in order to develop strategies to ameliorate liver injury caused by viral hepatitis and slow down the disease progression.

HIF-1α and HIF-2α stabilization has been observed in HBV- or HCV-infected cells and in liver biopsies from patients with chronic viral hepatitis [7, 9, 115]. Moreover, HIF-1α polymorphisms are significantly associated with the development of HBV-related HCC [116]. These observations suggest a role for HIFs in the pathogenesis of viral hepatitis and/or its progression to liver cirrhosis and HCC.

Numerous studies have demonstrated that HBV and HCV can promote HIF protein stability and transcriptional activity under normoxia. The hepatitis B viral X protein (HBx), which is indispensable for viral replication, can directly interact with the bHLH/PAS domain of HIFα subunit, thereby decreasing its binding with pVHL and preventing degradation [117]. The carboxy terminus of HBx was found to be critical in activating HIFs [118]. Aside from directly stabilizing HIFs, HBx induces HDAC1 and metastasis-associated protein 1, which inhibit HIF-1α association with PHDs and thus stabilize HIFs [119]. Moreover, HBx is reported to activate MAPK pathway and in turn activate HIF-1α [120]. HCV-induced HIF stabilization is mainly due to HCV-induced oxidative stress and calcium signaling, which cause the activation of NF-κB and STAT3 [12].

Evidence suggests that HIFs may directly promote viral replication. It has been reported that HCV replication is augmented under hypoxia, and that inhibition of HIF-1α suppresses viral replication [14, 121]. HIFs may promote viral transmission and replication in hepatocytes through modifying cell permeability and energy metabolism. It is reported that HIF-induced VEGF production depolarizes hepatocytes and facilitates viral entry [122, 123]. HIF stabilization enhances cell survival, which indirectly promotes viral infection. HCV is known to impair mitochondrial oxidative phosphorylation; however, survival of infected cells is preserved by increased glycolysis, an adaptive response mediated by HIF-1α [115]. It is also reported that HIF-1α stabilization modulates tight junction proteins and promotes migration of HCV-infected cells [14].

Although some experimental data support a role for HIFs in viral replication and cellular responses to viral infection, the transcriptional targets involved remain to be elucidated. Aside from a role in viral hepatitis, HIFs are likely involved in the disease progression. HBx is strongly implicated in promoting angiogenesis, which is critical in fibrosis and cancer development. This effect of HBx is mediated by its induction of HIF stabilization and transcriptional activation of angiogenic factors such as VEGF [9]. Moreover, HIF-induced adenosine production and its signaling may impede host anti-viral immune responses, owing to the anti-inflammatory and immunosuppressive effects of adenosine [124, 125]. These observations suggest that HIF may serve as an important therapeutic target to inhibit viral replication, boosting anti-viral immunity, and curbing the progression of viral hepatitis to more severe forms of liver disease.

### HIF and hepatocellular carcinoma (HCC)

HCC is one of the most common cancers with more than half a million new cases occurring worldwide each year. Because of its poor prognosis and lack of treatment options, HCC ranks as the fifth most common cause of death in men and the ninth in women [126]. The majority of the HCC cases (>80 %) are associated with chronic hepatitis B and C infection occurring in less developed countries of East Asia and sub-Saharan Africa [127, 128]. However, in the past 20 years, the incidence of HCC in developed countries has been increasing. For example, the annual incidence in the US has increased about 80 % in recent years [129]. HCC arises from liver cirrhosis, which is progressed from chronic liver diseases, including NAFLD, ALD, and viral hepatitis. Clinical observations reveal that liver cirrhosis is present in nearly 80 % of patients with HCC, representing the most important risk factor for HCC development [98, 99]. Current treatment options for HCC are very limited. A small number of patients (15 %) with early stage of the disease may be cured by liver resection or transplantation. However, in about half of the patients, tumor recurrence and metastases occur post-resection [130, 131]. The majority of the patients present with advanced HCC, and it is refractory to chemotherapy. A multikinase inhibitor, sorafenib, is currently the only first-line chemotherapy that has been shown to improve overall survival for approximately 3 months. Therefore, it is important to better understand the pathogenesis of HCC in order to develop novel therapeutic strategies.

Both HIF-1α and HIF-2α have been observed to be expressed at higher levels in HCC tissues than those in matched, non-tumor-surrounding tissues [5, 8, 132]. The high expression levels of HIF-1α or HIF-2α have been correlated with worse tumor grade, venous invasion, intrahepatic metastasis, and capsule infiltration [8, 133, 134]. HIF-1α and/or HIF-2α stabilization is a poor prognostic marker, as it is associated with shorter disease free period and lower overall survival rate [135–137]. Furthermore, genetic variations of HIF-1α have been associated with higher risk of development and prognosis of HCC and could serve as biomarkers [138, 139]. These clinical observations strongly suggest a pathological role of HIFs in HCC development and progression.

Multiple factors contribute to the stabilization of HIFs during HCC development. HCC typically arises in the condition of cirrhosis with reduced vascularization, which leads to hypoxia and HIF stabilization [140]. HIFs can also be activated by HBV and HCV, which are major causes of HCC. In support of this, in the majority of tumor specimens from HBV-related HCC patients, increased expression of HIF-1α was observed and the level correlates with the expression of HBx [141]. The mechanistic involvement of HIFs in HCC development stems from their gene transcriptional activities. Many HIF target genes play critical role in the key processes of tumorigenesis, such as cell proliferation, glucose metabolism, angiogenesis, invasion, and metastasis [142, 143]. HIF-1α induces growth factors, including TGFα and IGF-2, which promote cell proliferation and survival [144]. HIF-1α directly binds to the promoter of Forkhead box M1 (FoxM1), a transcription factor that promotes proliferation in HCC [145]. The role of HIF-2α in HCC is less clear, and there have been conflicting reports. Some studies show that HIF-2α expression correlates with the progression of HCC [133]. However, recent evidence also supports a possible tumor suppressor role for HIF-2α in HCC [146, 147].

Metabolic re-progamming from oxidative phosphorylation to aerobic glycolysis, termed the Warburg effect, is a common characteristic and survival mechanism of cancer cells. Key enzymes that contribute to the Warburg effect, such as PDK1 and lactate dehydrogenase A (LDHA), and the enzymes catalyzing glucose metabolism, such as phosphoglycerate kinase 1 (PGK1), hexokinase 1 and 2 (HK1/2), glyceraldehyde-3-phosphate dehydrogenase (GAPDH), phosphofructokinase (PFK), and the glucose transporters (GLUT-1 and GLUT-3), are all HIF-1α target genes [148–152]. Angiogenesis is critical for tumor growth. HIF-1α and HIF-2α transcriptionally activate VEGF, which is a potent angiogenic factor promoting endothelial cell proliferation, migration, and tube formation, especially in areas of hypoxia [153]. Aside from VEGF, HIF-1α also induces SDF-1, angiopoietin 2, placental growth factor, PDGF-B, and stem cell factor, all of which have proangiogenic activities [154, 155].

Tumor metastasis is the primary factor of poor prognosis in HCC. EMT is a critical step for tumor cells to acquire motility. HIF-1α and HIF-2α play important roles in EMT through downregulation of E-cadherin [132]. HIFs induce several genes that are E-cadherin repressors, such as Snail, Twist1, transcription factor 3 (TCF3), Zfhx1a, and Zfhx1b [132, 156–159]. In HBV- or HCV-positive HCC samples, a correlation between Snail and Twist induction and tumor metastasis has been established [160]. Moreover, HCV glycoproteins induce cancer cell migration via upregulation of Snail and Twist in an HIF-1α-dependent mechanism [14]. Extracellular matrix degradation by MMPs, such as MMP2 and MMP9, is another key mechanism in tumor metastasis [161, 162]. HIF-1α is an important transcription factor in upregulating the expression of MMPs [161, 163, 164]. It has been reported that HIF-1 silencing by siRNA reduces MMP2 and MMP9, thereby inhibiting migration and invasion of malignant gliomas [165]. However, there is also evidence supporting that HIF stabilization may improve tumor vessel functions and reduce the risk of metastasis. For example, in mice with haplodeficiency of PHD2, in which HIF-2α is increased, the leakiness and distorted architecture of tumor vessels are improved [48]. The “normalized” vasculature is associated with reduced tumor invasiveness [48].

In summary, HIF stabilization and pathological contribution to HCC tumorigenesis have been demonstrated by numerous studies. The current evidence highlights the potential of inhibiting HIF as a therapeutic approach to treat HCC. Targeting HIF can be achieved by anti-sense oligonucleotides or small molecule inhibitors that promote HIF degradation or blocking HIF transcriptional activities. These strategies are currently being explored in both preclinical and clinical studies [166–169].

### HIF modifiers as therapies to treat liver diseases

**Fig. 3:**
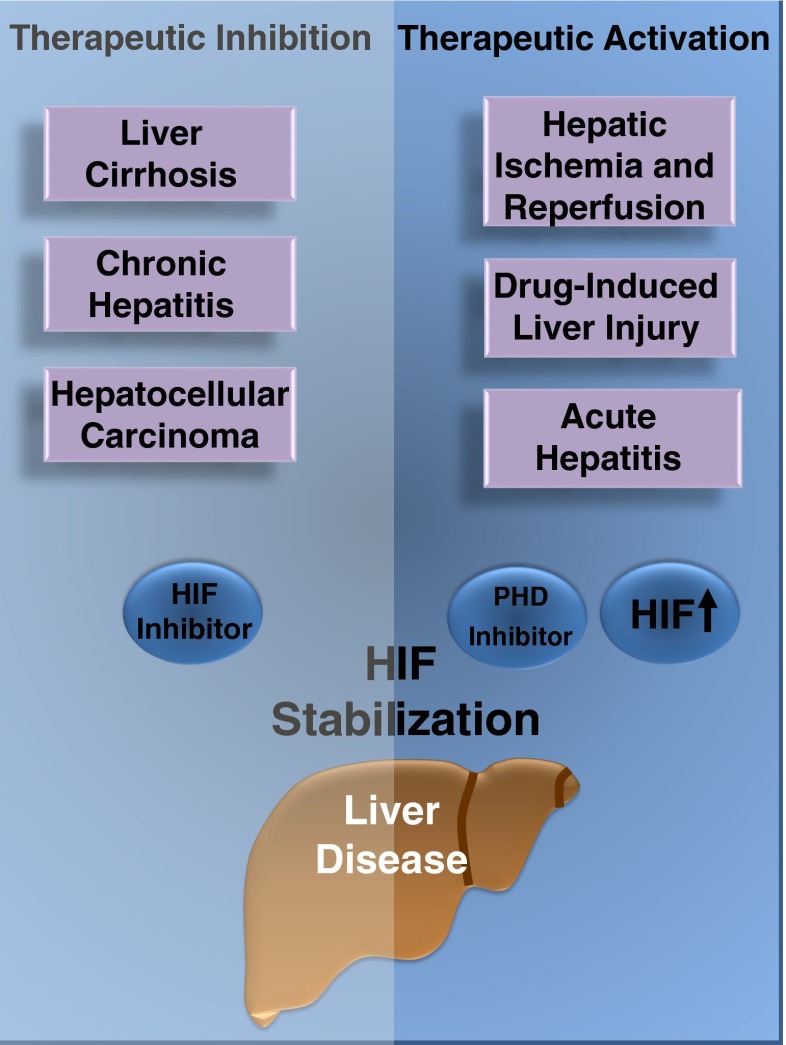
Targeting hepatic hypoxia-signaling for the treatment of liver disease. Most evidence points towards a tissue protective role of hypoxia-inducible transcription factors (HIFs) during acute liver injury, while in conditions of chronic liver disease, inhibition of HIFs may be desirable. Pharmacologic approaches to enhance hepatic stabilization of HIFs during liver disease can be achieved by pharmacologic inhibitors of prolyl hydroxylases (PHDs). Such compounds are currently examined in clinical trials and could potentially provide liver protection during acute liver injury. Clinical trials providing direct evidence for this hypothesis have yet to be completed and published. During conditions of chronic liver disease, inhibition of HIF stabilization in the liver may provide a therapeutic benefit. For example, oligo-nucleotide inhibitors for HIF are in clinical trials for the treatment of liver cancer

HIF has emerged as an important player in various liver diseases. Evidence supports a hepato-protective function of HIFs in acute liver injury, but a pathological role of HIFs in chronic liver disease. Although it appears to be paradoxical, the opposite involvement of HIFs in acute versus chronic liver diseases is consistent with their transcriptional activities. The target genes regulated by HIFs mediate adaptive responses to tissue stress and damage; however, the same genes that promote cell survival and proliferation and tissue repair lead to detrimental effects in chronic situations. Therefore, targeting HIF for therapies require very different approaches (Fig. 3). In situations of acute liver damage, such as IR liver injury, drug-induced liver failure, and acute hepatitis, HIF stabilization is desirable. Pharmacological inhibitors of PHDs, which stabilize HIFs, are currently being explored as treatment for ischemia, acute kidney, lung, or heart injuries. For example, clinical trials are ongoing to evaluate the safety and efficacy of several PHD inhibitors, such as FG-4592, AKB-6548, GSK-1278863, in the treatment of anemia in patients with chronic kidney disease, cardiac ischemia, and those requiring dialysis [60]. These inhibitors may be beneficial in attenuating acute liver injury. For example, to prevent IR injury, HIF activators may be administered to liver donors prior to explantation of the liver or added into the perfusate of the explanted liver. Moreover, it has been reported that HIF is stabilized and contributes to liver regeneration through facilitating gluconeogenesis and promoting liver progenitor cell proliferation [170–172]. Thus, a beneficial effect of HIF stabilizers on major liver resection warrants investigation. During phase II clinical trial of a PHD inhibitor, FG-2216, hepatotoxicity was identified and determined to be an off-target effect. A second-generation PHD inhibitor from FibroGen, FG-4592 was found to be safe during phase II and III clinical trials, and hepatotoxicity has not been reported for other PHD inhibitors in clinical trials. Moreover, based on the mechanism of action of the hydroxylase inhibitors and the evidence for the pro-survival effects of PHD inhibition and HIF stabilization, it is not likely that hepatotoxicity is a common side effect of this class of compounds [60].

In cases of chronic liver disease, cirrhosis and HCC in particular, inhibition of HIF may prove to be an effective treatment. In fact, targeting HIFs is being explored to treat HCC (examples are listed in Table 1). A HIF-1α antisense oligonucleotide, EZN2968, has undergone phase I clinical trial in advanced malignancies. It reduced tumor size without improving clinical efficacy. This HIF-1α antisense antagonist (under the name RO7070179) is now undergoing a phase 1b trial in HCC patients who have failed at least one line of systemic therapy [168]. A fusion protein containing Gal4 and the C-terminal 41 residues of HIF-1α transactivation domain (TAD-C) is reported to block the interaction of HIF-1α with its co-activator p300/CBP. In xenograft models, this fusion protein attenuates expression of HIF-1α target genes and reduces tumor growth [167]. Moreover, because of the involvement of HIFs in developing resistance to chemotherapy in HCC [173–178], HIF inhibitors can be given in combination with current therapies. For example, it has been shown that resistance to sorafenib can be overcome by EF24, which induces HIF-1α degradation [179]. In vitro and in vivo studies reveal that knock-down of HIF-2α with shRNA enhances the efficacy of sorafenib. [173, 178] Another study shows that treatment of antisense HIF-1α synergizes with doxorubicin in suppressing tumor growth and angiogenesis [180]. Overall, preclinical studies strongly suggest HIF inhibition as a strategy to treat chronic liver disease, HCC in particular. However, cautions need to be taken in clinical studies. For example, it may be important to discontinue HIF inhibitor therapies in patients who are undergoing liver surgery for HCC, as HIF inhibitors have the potential to exacerbate IR injury and inhibit liver regeneration.

### Concluding remarks

HIF stabilization has been reported in animal models and clinical samples of various liver diseases. The functional consequences of HIF stabilization in liver diseases are beginning to be elucidated. Current evidence supports a hepato-protective function of HIFs in acute liver damage. In this case, the HIF transcriptional activities cause protection of cells, promotion of angiogenesis, and reprogramming of cellular energy metabolism. However, the same functions of HIFs result in a pathological role in hepatic lipid accumulation, fibrogenesis, and tumor progression during chronic liver diseases. These findings indicate that HIF activation is desirable in acute liver injury, but HIF inhibition can prevent and ameliorate chronic liver disease. The dichotomous role of HIFs indicates that the clinical effects of HIF modifiers may be time-sensitive and disease-dependent. For example, in HCC patients undergoing resection or liver transplantation, temporarily halting the use of HIF inhibitors but instead activating HIFs may be beneficial. The same strategy may be considered in liver cirrhosis patients suffering from an episode of acute-on-chronic liver injury.

With the emerging appreciation of the involvement of HIFs in various liver diseases, a challenge for basic research is to elucidate the underlying molecular mechanisms. It is important to uncover the transcriptional targets and signaling pathways that mediate the functions of HIFs in experimental models. Aside from hepatocytes, multiple cell types in the liver contribute to disease development. Therefore, it is also critical to investigate the protective or pathological role of cell-specific HIFs in various liver diseases. The knowledge gained from these studies will unravel the complex involvement of HIFs in liver disease and help the development of therapeutic strategies to modulate HIFs. HIF modifiers are actively being developed and evaluated clinically in treating ischemia and cancer. Although some of these compounds are undergoing clinical trials for liver transplantation and HCC, translational research endeavor should be further emphasized in the near future.
